# Prostate cancer genomics and racial health disparity

**DOI:** 10.18632/oncotarget.26399

**Published:** 2018-11-30

**Authors:** Vanessa M. Hayes, Weerachai Jaratlerdsiri, M.S. Riana Bornman

**Affiliations:** Laboratory for Human Comparative and Prostate Cancer Genomics, Genomics and Epigenetics Division, Garvan Institute of Medical Research, Darlinghurst, NSW, Australia; St Vincent's Clinical School, University of New South Wales, Randwick, NSW, Australia; Central Clinical School, University of Sydney, Camperdown, NSW, Australia; School of Health Systems and Public Health, University of Pretoria, Pretoria, South Africa

**Keywords:** prostate cancer, health disparity, African ancestry, genomics

Prostate cancer incidence and mortality rates are significantly associated with race or ethnicity. In the United States, men of African ancestry are at 1.6- to 2.9-fold increased risk for diagnosis and 2.4- to 5.0-fold increased risk of death from prostate cancer than men of European and Asian ancestry, respectively [1]. With no known modifiable risk factors and limited success in identifying significant socioeconomic or lifestyle contributors, a link between genetics and health disparity cannot be ignored. Studies focused on inherited genetic risk using genome-wide association analysis report African-specific risk-alleles [2]. However, studies focused on the acquired genomic landscape of African ancestral prostate tumor evolution have been scarce.

Genome profiling has identified prostate tumors as highly heterogenous. In contrast to other cancers, small coding somatic mutations are relatively scarce, while recurrent non-coding aberrations and larger genomic rearrangements, including E26 transformation-specific (*ETS*) family transcription factor gene fusions and somatic copy number alterations (SCNAs), are more common [3, 4]. Consequently, exome sequencing is regarded as having limited appeal, with consortia such as the Pan Prostate Cancer Group (PPCG) advocating for whole genome sequencing (WGS). We found a single study to have used WGS (specifically Complete Genomics) to interrogate the genomic landscape of Gleason score ≥ 6 tumors from 24 African American men [5], while data for Africa was lacking.

We have previously highlighted the untapped potential that Africa holds to unravel the genetic basis and evolution of prostate cancer, in particular aggressive disease [6]. Establishing in 2008 the Southern African Prostate Cancer Study (SAPCS), we observed a 2.1-fold increase in aggressive disease presentation in Black South Africans compared with age-adjusted African Americans [7]. To unravel the genetic basis for this disparity, we performed fresh-tumor and normal-blood paired WGS for six African (South African) and nine European (Australian) men presenting with treatment-naïve high-risk (Gleason score ≥ 8) prostate cancer [8]. Although a pilot analysis, direct race/ethic comparison allowed for limitations associated with between study comparisons, including sample processing, sequencing and data analyses, to be eliminated.

Tumor mutational burden (TMB), defined as the number of small substitutions acquired per megabase pair (Mbp), was significantly elevated within our African tumors. Excluding for a single uniquely hypermutated tumour, 55 mutations/Mbp, we observed a TMB of 3.8 (range 3.0 to 4.7) in our African *versus* 2.1 (range 1.7 to 2.8) in our European tumors, equating to a 1.8-fold increase (*P* = 1.02e-04). Compared with the most recent non-African WGS prostate tumour data [4], we observed a 3.8-fold greater TMB than reported for primary tumors (all grades), and 2.4- and 1.3-fold greater than reported for treatment-naïve and hormone-treated metastatic tumors, respectively. Restricting our analysis to exonic regions, we observed similar frequencies for protein coding, deleterious and oncogenic somatic mutations for the three high-risk TCGA-derived African American tumors, which was significantly elevated compared with pathologically-matched European-derived tumors. Within our study, single base substitution oncogenic driver mutations were significantly elevated in our African *versus* European tumors (median 17 *vs*. 6, *P* = 2.92e-03).

The importance of elevated TMB in our African tumors raises two novel hypotheses. Firstly, it is well established that TMB increases with carcinogenic impact [9]. One may therefore speculate that an as yet unknown carcinogen may be contributing to aggressive prostate cancer presentation within Africa. Again, Africa offers unique opportunities to address potential unknown environmental contributors [6]. Epidemiological risk associations identified within the SAPCS could be explained by regional specific exposures [10]. Secondly, increased TMB has been associated with a favorable response to immunotherapeutics [11]. While prostate cancer has been less responsive to immunotherapy, increased TMB within African patients may increase immunotherapeutic response rates within patients of African heritage.

Other notable African-specific differences included the predominance of cancer mutational signatures of unknown underlying processes (specifically signatures 5, 8 and unidentified), which again supports the notion of an as yet unknown carcinogenic agent. Our African tumors lacked *ETS* fusions and larger genomic rearrangements were under-represented, including chromothripsis- and chromoplexy-like events. In contrast and as previously reported, roughly half of the European-derived tumors presented as *ETS*-positive (*ERG* or *ETV1*), with associated chromoplexy. Wedge et al, [4] reported an increase in genomic rearrangements in metastatic over primary prostate cancer, largely driven by hormone treatment, with no differences in chromoplexy. Unique to this study, we observed a single hyperduplicated treatment-naïve African-derived tumor with 234 tandem duplications implicating 149 transposable elements. Additionally, we observed a higher overall acquisition of copy number losses and gains.

We found no overlap between the 14 genes identified in our African cohort with recurrent non-synonymous putative oncogenic driver mutations and the most recent published list of 73 prostate cancer driver genes [4]. However, of these 73 driver genes 19 usual prostate cancer gene candidates were mutated in at least one African tumor, while non-coding *FOXA1* driver mutations were reported by both studies. We speculate that prostate cancer driver events may be race-specific and lead to health disparity, while others are likely cancer-specific or race-agnostic. Additional African-specific signatures included, an increase in SCNAs in late stages of tumor evolution (sub-clonal, *P* = 0.03), notably all presenting with gains at 1p36.13, 4p16.3, and 9q13, while the predominance of small somatic substitutions early in tumorigenesis, raises potential for early diagnosis and prognosis.

It is well established that germline variation contributes as much as 58% to overall prostate cancer risk [12], yet identifying high-penetrance genes that may explain the observed health disparity has been challenging. Reporting a 1.3-fold increase in germline variation in our African *versus* European patients, no pathogenic mutations were found in known high penetrance genes, such as *BRCA1, BRCA2, ATM, HOXB13,* and *CHK2*. We speculate that as yet unidentified genes may be contributing to increased aggressive disease presentation within Africa, calling for further inclusion of African cohorts in current association studies.

In conclusion, our study highlights significant difference in the genomic landscape underpinning high-risk prostate cancer in African compared with non- African derived prostate tumorigenesis. Calling for further African-derived genome profiling, our study provides optimism for new opportunities and potential for clinical management of prostate cancer within Africa.
